# Small pre-trained model for background understanding in multi-round question answering

**DOI:** 10.3389/frai.2024.1308206

**Published:** 2025-05-30

**Authors:** Xin Huang, Hulin Song, Mingming Lu

**Affiliations:** ^1^Software College, Jiangxi Normal University, Nanchang, China; ^2^School of International Economics and Trade, Jiangxi University of Finance and Economics, Nanchang, China; ^3^Department of Computer Science and Technology, Tongji University, Shanghai, China

**Keywords:** multi-round Q&A, knowledge transfer, background understanding, knowledge distillation, model compression

## Abstract

Multi-round Q&A based on background text needs to infer the answer to the question through the current question, historical Q&A pairs, and background text. The pre-trained model has proved its effectiveness in this task; however, the existing model has many problems such as too many parameters and high resource consumption. We propose a knowledge transfer method that combines knowledge distillation, co-learning of similar datasets, and fine-tuning of similar tasks. Through multi-knowledge cooperative training from large model to small model, between different data sets, and between different tasks, the performance of the small model with low resource consumption can match or surpass that of the large model.

## 1 Introduction

Background-oriented text question answering (Q&A) studies (Minaee et al., 2021; Li et al., 2022; Cui et al., 2022; Huang et al., 2023b) derived from machine-reading comprehension tasks represented by SQuAD (Rajpurkar et al., 2016, 2018) are gaining attention. Considering a background text fragment and a question associated with it, we try to determine an answer to a question based on the background text or mark that the answer does not exist. Although multiple issues with the background text exist, none of them are related. However, in a real environment, Q&A is a multi-round and continuous process, and the questions are not independent as students may ask questions to teachers on random topics of interest (Stede and Schlangen, 2004; Huang et al., 2023c). Because numerous coreferences and ellipses are used in multi-round of Q&A to achieve concision and efficiency, question comprehension should consider the current question content and combine historical Q&A pairs to determine the object of pronoun reference and ellipsis content to understand the current question in detail. The challenge of background comprehension in multi-round Q&A is the deduction of an answer to a question based on the current question, historical Q&A pairs, and background text (Martinez-Gil, 2023; Cui et al., 2023; Shao et al., 2023).

To promote studies on multi-round Q&A considering background and evaluate the validity of the Q&A model, a few datasets have been published, such as CoQA (Reddy et al., 2019) and QuAC (Choi et al., 2018). Each of these datasets involves multiple rounds of Q&A around a single piece of background text; both datasets include two speakers (questioner and responder) and allow for unanswerable questions. Table 1 lists a complete multi-round Q&A based on the CoQA dataset. The background relates to a CNN news report divided into two sections. According to the conversion method proposed by Yatskar (2019), CoQA answers can be converted into four types: (1) SPAN representing the background text fragment, (2) affirmative YES, (3) negative NO, and (4) UNANS that cannot be answered.

**Table 1:** Q&A example of CoQA.

**(CNN)–American journalist Michael Scott Moore, held for more than 2 years by Somali pirates, has been freed, Moore's family and a Somali official told CNN on Tuesday**.	
“We are just elated,” Marlis Saunders, Moore's mother, said in a brief conversation. “It took a lot of work for us to get this point. And to hear he is free—just joyful, I can't describe it”	
*Q*_1_: Who was held for two years?	*Q*_6_: Who?
*A*_1_: Michael Scott Moore	*A*_6_: Marlis Saunders
*Q*_2_: What happened to him?	*Q*_7_: Who was that?
*A*_2_: freed	*A*_7_: Moore's mother
*Q*_3_: From who?	*Q*_8_: How Did she feel?
*A*_3_: Somali pirates	*A*_8_: elated
*Q*_4_: After how long?	*Q*_9_: Why?
*A*_4_: More than 2 years	*A*_9_: To hear he is free
*Q*_5_: Did anyone feel about this?	*Q*_10_: How does she describe it?
*A*_5_: Yes	*A*_10_: She can't.

With an increase in the number of pre-training models (Qiu et al., 2020; Yu et al., 2022; Gou et al., 2021), corresponding studies have introduced pre-training models for new studies (Singh et al., 2021; Gu et al., 2021; Kandpal et al., 2023; Lauriola et al., 2022). From the beginning, it was a supplement to the traditional word vector (Yatskar, 2019), which is regarded as a downstream task of the pre-training model, and the model performance was improved by adding word-embedding information (Qu et al., 2019), adjusting the output layer structure (Yeh and Chen, 2019), and improving the training objectives (Ju et al., 2019; Garg and Moschitti, 2021; McCarley et al., 2020; Chen et al., 2021; Yang et al., 2020). We explored a small pre-training model suitable for Q&A background awareness to ensure that the performance of the small model matches or surpasses that of the large model and achieves an effective environment of resource consumption and performance. We proposed a model based on knowledge transfer—KTM. Knowledge transfer is divided into two stages: “preparation” and “learning.” First, three fine-tuned large and small models were obtained during the preparation phase. The fine-tuning of the small model was performed on the machine-reading comprehension dataset, that is, SQuAD, which allowed the small model to learn knowledge on similar tasks. Next, in the learning stage, knowledge distillation was applied, and CoQA and QuAC datasets were combined to learn together. The small model learns the knowledge owned by the large model; in contrast, the two background understanding datasets learn from each other and complement each other. Comparative experiments show that compared with the large model, KTM has fewer parameters, lower memory usage, significantly shorter training time, and faster prediction speed, while maintaining the excellent background understanding ability of the large model. The contributions of this study are as follows.

1. The computational time and performance differences in the background understanding task of the automatic Q&A of mainstream pre-training models were compared.

2. A small-scale pre-training model for the background understanding of Q&A was proposed. Knowledge distillation, co-learning of similar datasets, fine-tuning of similar tasks, and other strategies were comprehensively used to achieve various types of knowledge transfers to ensure that the performance of small models with low resource consumption matched or exceeded that of large models.

3. Abundant validation experiments were performed to demonstrate the effectiveness of knowledge transfer. Experimental results on the QuAC validation set indicate that the performance of the knowledge transfer model exceeds that of the large model.

## 2 Related work

While the foundational work by Hinton et al. (2015) on knowledge distillation provides a basic framework, recent studies have applied knowledge distillation techniques specifically within QA systems (Gou et al., 2021). For instance, Izacard and Grave (2020) demonstrated how distilling knowledge from a reader to a retriever enhances the efficiency of open-domain QA systems. Moreover, Yang et al. (2019b) focused on model compression within large-scale QA systems through multi-task knowledge distillation. Unlike these studies, our approach uniquely optimizes small model performance in a multi-round QA setting by integrating knowledge distillation with co-learning, a combination less explored in prior research.

Considering model performance and computing resources, the traditional background understanding model represented by BiDAF++ w/ *n*-ctx demonstrates lower memory consumption, short training and prediction time, and poor performance. The pre-training model can considerably improve the performance of background understanding; however, its large number of parameters increases resource consumption and renders it difficult to deploy. In addition, the parameters of large and small models are not proportional to performance. For example, XLNet (Yang et al., 2019a) has numerous parameters but does not perform as well as ALBERT (Lan et al., 2020). The current trend of pursuing large models is worrisome for the environment (Schwartz et al., 2019), and performance can be improved only by consuming a large number of computing resources (Wu et al., 2022; Chang et al., 2022; Huang et al., 2023a).

Table 2 compares the application of different models to the CoQA dataset from three aspects: parameter quantity, computation time, and performance. Among the five pre-training models, BERT (Devlin et al., 2019) was first proposed, and the following four models, XLNet, RoBERTa (Liu et al., 2019), ALBERT, and DistilBERT (Sanh et al., 2019), are all improvements to BERT.

**Table 2:** Trainable parameters of traditional and pre-trained models in CoQA.

**Model**	**Trainable**	**Round**	**Predict**	**Validation**
	**Param**	**(h)**	**(min)**	**F1**
**BiDAF++ w/** *n***-ctx**	**2M**	**1.12**	**5.75**	**69.2**
BERT-base	110M	11.17	16.33	80.5
XLNet-base	117M	50.08	23.67	78.8
RoBERTa-base	125M	11.42	16.17	80.2
ALBERT-base	12M	10.67	10.18	80.6
DistilBERT-base	66M	5.50	8.02	75.0

The training and prediction time is the time required to complete the corresponding operation of the model considering a single Tesla K40m graphics card. Among them, Round (h) represents the time required for one round of training, in hours, and Predict (min) represents the time required for prediction, in minutes.

1. The parameter quantity of BiDAF++ w/ *n*-ctx is much smaller than that of the pre-trained model; the former is ~1/10 of the latter. As lightweight pre-training models, ALBERT-base and DistilBERT-base have 90 and 40% fewer parameters than BERT-base, respectively.

2. BiDAF++ w/ *n*-ctx consumes less time than the pre-training model owing to its smaller number of parameters, and the maximum difference is ~50 times. Remarkably, the training time of ALBERT-base was close to that of BERT-base. This implies that the smaller the number of parameters, the smaller the time consumption; however, ALBERT-base does not conform to this rule. ALBERT reduces the number of parameters but not the number of calculations. DistilBERT-base complies with the aforementioned rules, and its training and prediction times are reduced by 50%, which is aligned with the reduction in the number of transformer coding layers by 50%.

3. In terms of performance, the advantages of pre trained models have been fully demonstrated. The F1 values of BERT-base, RoBERTa-base, and ALBERT-base differ by more than 10 compared to BiDAF++ w/ *n*-ctx. Even DistiBERT base, which has the worst performance among the five pre-trained models, has an F1 value difference of 5.8.

In summary, the larger the model, the better the balance between deep model performance and resource consumption, which is the aim of this study.

## 3 Approach

### 3.1 Task definition

Given a background text of length m *B* = {*b*_1_, *b*_2_, …, *b*_*m*_}, current question *Q*_*i*_ (*i* ⩾ 1), and historical Q&A pair {*Q*_1_; *A*_1_; *Q*_2_; *A*_2_; …;*Q*_*i*−1_; *A*_*i*−1_}, the goal of the background understanding of the Q&A system is to generate the answer *A*_*i*_ of the question *Q*_*i*_, which requires the type of *A*_*i*_ *t* to be SPAN, YES, NO, or UNANS. In particular, the SPAN type requires that the answer must be a span of the background text, that is, $A_{i}={\left\{b_{j}\right\}}_{j=k}^{l}$(1 ⩽ *k* ⩽ *l* ⩽ *m*), and must satisfy *l* − *k* ⩽ *n*, *n* is the maximum allowed length of the answer, and different datasets have different values. If the answer is to the other three types, *A*_*i*_ is determined by the dataset. For example, UNANS answers are represented by “unknown” and “CANNOTANSWER” in CoQA and QuAC, respectively.

### 3.2 Pre-trained models in background understanding

**Figure 1:**
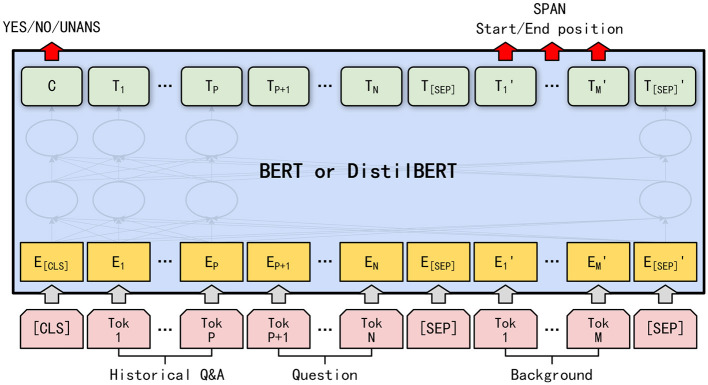
Background understanding based on pre-trained models.

Figure 1 shows the background understanding model based on BERT or DistilBERT. As the main difference between BERT and DistilBERT is the number of transformer coding layers, and there is no difference between the input and output, a uniform purple box is used to represent the two models in the figure, which ignores differences in the internal structure of the models.

The input to the model comprises two concatenated sequences: *s*_1_ and *s*_2_ of length *N* and *M*, respectively. Sequence *s*_1_ includes two parts: the historical Q&A pair and current question.


(1) $$\begin{array}{l} s_{1}=\left\{\text{[Q]};Q_{1};\text{[A]};A_{1};\ldots ;\text{[Q]};Q_{i-1};\text{[A]};A_{i-1};\text{[Q]};Q_{i}\right\}, \end{array}$$

where [Q] and [A] are special words that mark question and answer sentences, respectively, and are located at the beginning of the sentence. As question *Q*_1_ has no historical Q&A pairs, *s*_1_ = {[Q];*Q*_1_}. Sequence *s*_2_ includes background *B*.


(2) $$\begin{array}{l} s_{2}=B. \end{array}$$

The two sequences are spliced together before inputting BERT or DistilBERT: {[CLS];*s*_1_; [SEP]; *s*_2_; [SEP]}. Here, [CLS] is used to calculate the probability of the answer type, and [SEP] is used to segment the sequence. After entering the model, first, each word was converted into a vector, which was obtained by adding three parts: word embedding, segment embedding, and position embedding (DisitlBERT has no segment embedding). Segment embedding indicates whether the word belongs to *s*_1_ or *s*_2_, and positional embedding indicates the position of the word in the input sequence. Subsequently, after multi-layer transformer encoding, the latent vector of the last layer of each word was used as the output of the pre-training model, which was recorded as ***T*** ∈ ℝ^*d*×*L*^. Based on ***T***, we calculated the probability of the start position *k* and the end position *l* of the segment when the answer is of type SPAN, and the probability of the answer is of types YES, NO, and UNANS. In particular, the calculation methods for the probability distributions *p*^*k*^ and *p*^*l*^ ∈ ℝ^*L*^ of *k* and *l* are as follows.


(3) $$\begin{array}{l} p^{k}=softmax\left(w_{1}^{T}T+b_{1}\right),and \end{array}$$

(4) $$\begin{array}{l} p^{l}=softmax\left(w_{2}^{T}T+b_{2}\right), \end{array}$$

where ***w***_1_, $w_{2}\in {\mathbb{R}}^{d}$, *b*_1_, *b*_2_ ∈ ℝ are to-be-trained parameters. The probability distribution *p*^*t*^ ∈ ℝ^3^ of the three types of answers, YES, NO, and UNANS, is calculated as follows.


(5) $$\begin{array}{l} p^{t}=softmax\left(w_{4}^{T}\tanh\left(w_{3}^{T}C+b_{3}\right)+b_{4}\right), \end{array}$$

where ***C*** ∈ ℝ^*d*^ is the hidden vector of [CLS], and $w_{3}\in {\mathbb{R}}^{d\times d}$, $b_{3}\in {\mathbb{R}}^{d}$, $w_{4}\in {\mathbb{R}}^{d\times 3}$, $b_{4}\in {\mathbb{R}}^{3}$ are parameters to be trained. Finally, we determined the answer-type estimate $\hat{t}$ as follows.


(6) $$\begin{array}{l} p_{max}=\max\left(p^{k}+p^{l}\right),\;0⩽l-k⩽n, \end{array}$$

(7) $$\begin{array}{l} a=\arg\max p^{t}, \end{array}$$

(8) $$\begin{array}{l} \hat{t}=\{\begin{array}{ll} ANS_{a}, & \;\text{if }p_{1}^{k}+p_{1}^{l}>p_{max},\\ \text{SPAN}, & \;\text{else}. \end{array} \end{array}$$

In Equation 8, *ANS* = {YES, NO, UNANS}, $p_{1}^{k}$ and $p_{1}^{l}$ are the starting and ending position probabilities corresponding to [CLS], respectively. If the answer type is SPAN, its start and end position estimates $\left(\hat{k},\hat{l}\right)$ are Equation 6 when *k* and the value of *l*:


(9) $$\begin{array}{l} \left(\hat{k},\hat{l}\right)=\underset{k,l}{\arg\max}\left(p^{k}+p^{l}\right),\;0⩽l-k⩽n. \end{array}$$

The three probability distributions of Equations 3–5 are all output by Softmax; therefore, we used the sum of the negative logarithmic likelihood to construct the loss function L(θ) for background understanding:


(10) $$\begin{array}{l} L\left(\theta \right)=-\frac{1}{\;|D|\;}\underset{i}{\sum }[𝟙\left(y_{i}^{t}=\text{SPAN}\right)\left(\log p_{y_{i}^{k}}^{k}+\log p_{y_{i}^{l}}^{l}\right)\\ \;+𝟙\left(y_{i}^{t}\neq \text{SPAN}\right)\log p_{y_{i}^{t}}^{t}]. \end{array}$$

where θ is the model parameter, |D| is the number of training samples, 𝟙(·) is the indicator function, $y_{i}^{t}$ is the real type of answer *A*_*i*_, and $y_{i}^{k}$ and $y_{i}^{l}$ are the start and end positions of the span, respectively.

### 3.3 Knowledge transfer method

**Figure 2:**
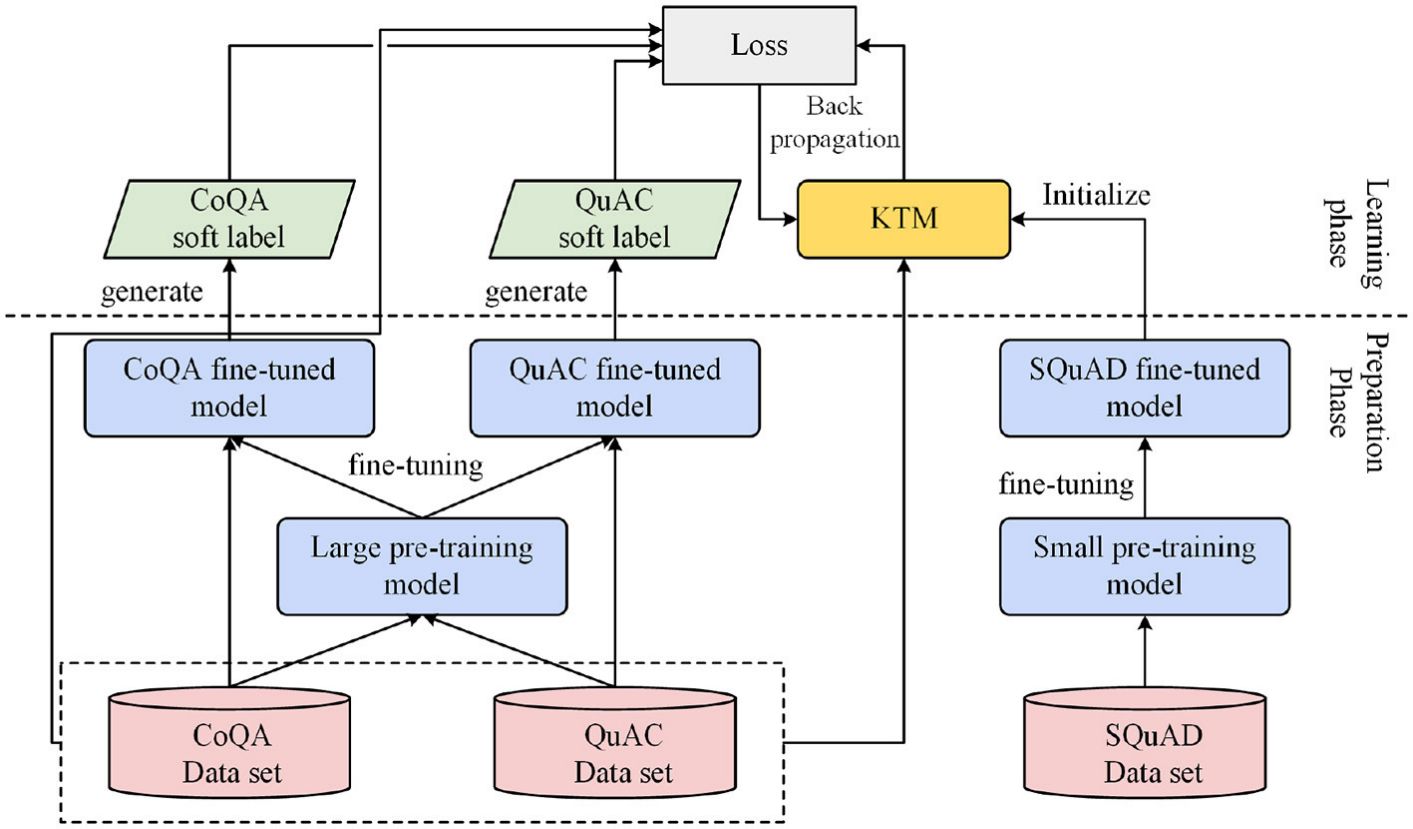
Knowledge transfer process for background understanding.

The size model is a relative concept determined by the number of parameters. A model with more parameters is called a large model, while a model with fewer parameters is called a small model. Specifically, in this article, the large model refers to BERT-base, while the small model is DistillBERT-base. Figure 2 shows the main steps and processes of the knowledge transfer method. First, we fine-tuned large and small models. The size of the model was determined based on the number of parameters. A model with a large number of parameters is known as a large model, whereas a model with a small number of parameters is known as a small model. The large and small models can be two with the same structure (e.g., BERT and DistilBERT) or two with different structures (e.g., BERT and BiDAF++ w/ *n*-ctx). In particular, fine-tuning was performed on two datasets, CoQA and QuAC, to obtain CoQA and QuAC fine-tuned large models, respectively. Small model fine-tuning was performed on the machine-reading comprehension SQuAD dataset to obtain SQuAD fine-tuned small models. Fine-tuning is used to prepare for subsequent work; therefore, this process is known as the preparation phase.

The next phase is learning, which is the core of the knowledge transfer method and is roughly divided into the following three steps. The first step is to initialize the KTM with a small model fine-tuned using SQuAD. The second step is to combine the training samples of the two datasets, CoQA and QuAC, and input them into the KTM and fine-tuned large models to generate the predicted values and soft labels of the samples, respectively. The third step is to calculate the loss value based on the predicted value and soft label, as well as the true label (hard label) of the sample; subsequently, let the gradient propagate back to the KTM. In the aforementioned steps, the method of combining datasets to learn is known as co-learning, and the method of using soft labels to calculate the loss value is known as knowledge distillation.

In this study, the purpose of SQuAD fine-tuning was to let KTM learn in advance how to extract answers from the background text and jointly learn the data characteristics shared between CoQA and QuAC by expanding the training samples. Knowledge distillation allows KTM to master answers learned from the big model.

Therefore, the proposed knowledge transfer method used SQuAD fine-tuning, co-learning, knowledge distillation, and knowledge transfer between different tasks, between different data sets, and from large to small models. The SQuAD fine-tuning method has been described in Devlin et al. (2019).

### 3.4 Knowledge distillation

**Figure 3:**
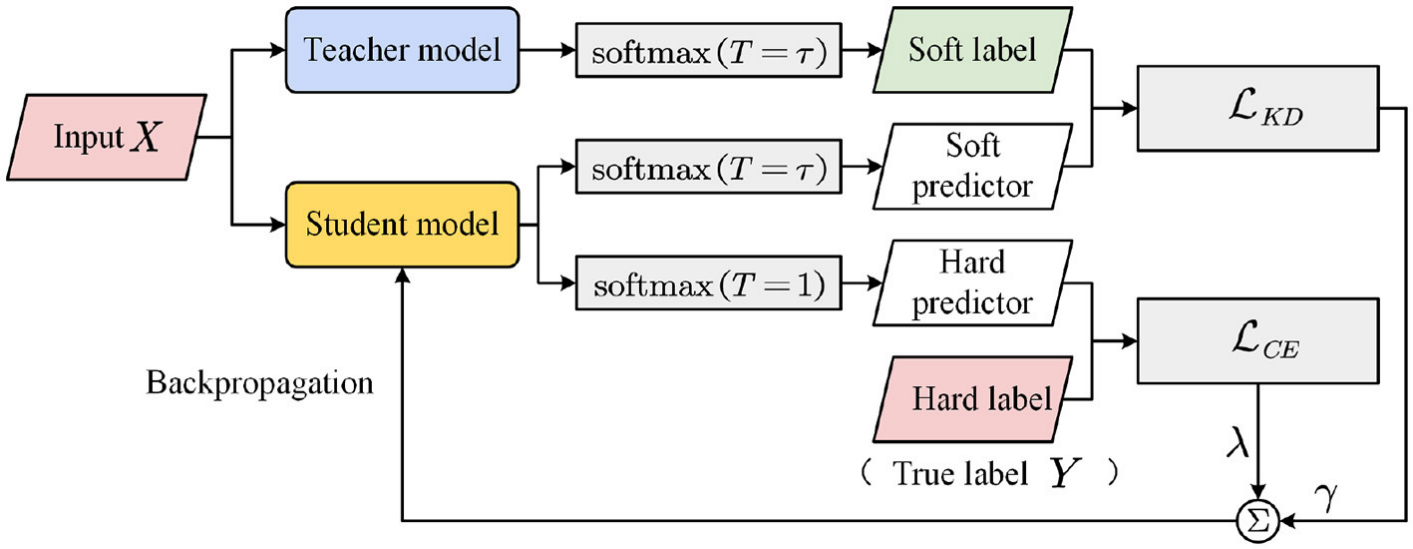
Knowledge distillation framework.

The knowledge distillation framework shown in Figure 3 contains two models: teacher and student. The teacher model is a large trained model or an ensemble of multiple models, whereas the student model is a small model that learns from the teacher model. The idea of knowledge distillation is to let the student model learn from ground-truth labels and the probability distribution output of the teacher model.

Given an *m* classification dataset of the form (*X, Y*), the classifier can be trained by minimizing the cross-entropy loss function L:


(11) $$\begin{array}{l} p=softmax\left(z\right), \end{array}$$

(12) $$\begin{array}{l} L_{CE}=-\overset{m}{\underset{k=1}{\sum }}Y_{k}\log p_{k}, \end{array}$$

where *p* is the class probability distribution, and ***z*** ∈ ℝ^*m*^ is the logits of the model. If the aforementioned training process is the process of classifier learning according to the real label *Y*, then knowledge distillation includes the process of student model learning according to the class probability distribution *q* output based on the teacher model. Similar to *Y*, in several cases, the probability value of *q* for the correct class will be high, approaching 1, and the probability of the other classes will be 0. Thus, *q* does not provide more information than *Y* and does not make much sense for model training. To solve this problem, Hinton et al. (2015) introduced the concept of temperature into the softmax function. The modified softmax function is


(13) $$\begin{array}{l} softmax{\left(z;T\right)}_{k}\equiv \frac{\exp\left(z_{k}/T\right)}{\;\sum_{j}\exp\left(z_{j}/T\right)\;}, \end{array}$$

where *T* denotes the temperature. When *T* = 1, Equation 13 reduces to the standard softmax function.

Applying the modified softmax function to the teacher and student models, we obtain


(14) $$\begin{array}{l} q=softmax\left(z_{t};T=\tau \right),and \end{array}$$

(15) $$\begin{array}{l} u=softmax\left(z_{s};T=\tau \right), \end{array}$$

where ***z***_*t*_ and ***z***_*s*_ are the logits of the teacher and student models, respectively, and τ is a hyperparameter. The larger the value of *T*, the more “soft” *q* and the more information the teacher model provides. Therefore, *q* is often known as a soft label, the corresponding real label *Y* is the hard label, *u* is the soft predicted value, and *p* is the hard predicted value. According to Equations 14, 15, the loss function $L_{KD}$ when the student model learns the output of the teacher model is defined as


(16) $$\begin{array}{l} L_{KD}=-\overset{m}{\underset{k=1}{\sum }}q_{k}\log u_{k}. \end{array}$$

The overall optimization objective of the student model is the weighted sum of the two loss functions $L_{CE}$ and $L_{KD}$.


(17) $$\begin{array}{l} \lambda L_{CE}+\gamma L_{KD}. \end{array}$$

where λ and γ are the weights.

### 3.5 Training method

Based on the knowledge distillation theory (Hinton et al., 2015), the proposed KTM was considered as the student model, and the two fine-tuned large models were considered as the teacher models. The CoQA soft label, QuAC soft label, and KTM soft prediction value are denoted by $q_{\text{CoQA}}^{*}$, $q_{\text{QuAC}}^{*}$, and u^*^ (* = *k, l, t*), respectively. Considering *u*^*k*^, the calculation method is defined as


(18) $$\begin{array}{l} u^{k}=softmax\left(w_{1}^{T}T+b_{1};T=\tau \right). \end{array}$$

According to the aforementioned soft labels and soft prediction values, the loss function of the KTM ($L_{KD}\left(\Theta \right)$) can be obtained as


(19) $$\begin{array}{r} L_{KD}\left(\Theta \right)=-\frac{1}{\;|D|\;}\underset{i}{\sum }[\overset{L}{\underset{j=1}{\sum }}\left(q_{j}^{k}\log u_{j}^{k}+q_{j}^{l}\log u_{j}^{l}\right)\\ +\overset{\left|ANS\right|}{\underset{a=1}{\sum }}q_{a}^{t}\log u_{a}^{t}], \end{array}$$

where Θ is the sum of the parameters of KTM, and |·| represents the number of set elements. In summary, the optimization goals of the KTM are


(20) $$\begin{array}{l} L\left(\Theta \right)=\lambda L_{CE}\left(\Theta \right)+\gamma L_{KD}\left(\Theta \right). \end{array}$$

In particular, QuAC only has two answer types, SPAN and UNANS, and the probability distribution *p*^*t*^ can be discarded. The answer type is determined to be UNANS according to $p_{0}^{k}+p_{0}^{l}>p_{max}$. Therefore, for the QuAC dataset, the estimation method for the answer type ${\hat{t}}_{\text{QuAC}}$ is as follows.


(21) $$\begin{array}{l} {\hat{t}}_{\text{QuAC}}=\{\begin{array}{ll} \text{UNANS}, & \text{if }p_{0}^{k}+p_{0}^{l}>p_{max},\\ \text{SPAN}, & \text{else}. \end{array} \end{array}$$

Accordingly, the optimization objective L(Θ) simplifies to


(22) $$\begin{array}{r} L_{\text{QuAC}}\left(\Theta \right)=-\frac{1}{\;|D|\;}\underset{i}{\sum }[\lambda \left(\log p_{y_{i}^{k}}^{k}+\log p_{y_{i}^{l}}^{l}\right)\\ +\gamma \overset{L}{\underset{j=1}{\sum }}\left(q_{j}^{k}\log u_{j}^{k}+q_{j}^{l}\log u_{j}^{l}\right)]. \end{array}$$

Based on the aforementioned optimization objectives, we developed the KTM training mechanism of the KTM, as explained in Algorithm 1. As the KTM training process involves two datasets, first, CoQA and QuAC, the mini-batches of the two datasets were merged, and the combined KTM training set is denoted as D. The entire training process iterated *epoch*_*max*_ rounds. Before each round, the training set D was disturbed to ensure the randomness of the training samples. Subsequently, a mini-batch *b*_α_ was selected from the out-of-order D with samples from the same dataset α. *B*_α_ was input into the large model fine-tuned by dataset α and KTM, and the soft labels and soft and hard predicted values needed to calculate the loss value were output. If α= CoQA, use Equation 20 to calculate the loss value; if α= QuAC, use Equation 22. Finally, the gradient of each parameter was calculated from the loss value, and the KTM was updated.

**Algorithm 1:**
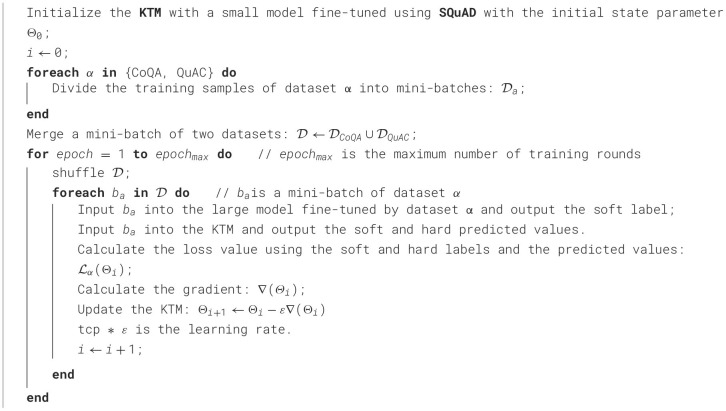
KTM training.

## 4 Experiment

### 4.1 Details

For datasets, CoQA and QuAC use release versions, whereas SQuAD uses version 2.0 (Rajpurkar et al., 2018). In terms of data preprocessing, all characters were lowercased, the maximum length of *s*_1_ was maximum (64), and the sliding step size was stride = 128. The dropout probability of each layer was 0.1, the softmax temperature was τ= 2, and the loss function weights were λ= 0.5 and γ= 0.5. For training, the mini-batch size was 12, and the maximum number of iterations was *epoch*_*max*_= 2. The optimizer was Adam (Kingma and Ba, 2014), and the learning rate was ϵ= 3e-5. The first 10% of the training samples were used to warm up the learning rate and then decay linearly. In terms of prediction, the maximum answer length *n* of the two datasets was different: 17 for CoQA and 30 for QuAC.

### 4.2 Metrics

CoQA and QuAC use word level F1 as the main metric, which is calculated as follows.


(23) $$\begin{array}{l} overlap=|S_{pred}\cap S_{gold}|, \end{array}$$

(24) $$\begin{array}{l} P=\frac{\;overlap\;}{\left|S_{pred}\right|}, \end{array}$$

(25) $$\begin{array}{l} R=\frac{\;overlap\;}{\left|S_{gold}\right|}, \end{array}$$

(26) $$\begin{array}{l} F_{1}=\frac{\;2\times P\times R\;}{P+R}, \end{array}$$

where *S*_*pred*_ and *S*_*gold*_ are the sequences of the predicted and standard answers, respectively, and |·| is the length of the sequence. We combined the two-word sequences, considered the overlapping part, calculated its length as *overlap*, and divided it with the predicted answer length and standard answer length, respectively. The accuracy *P* and recall rate *R* were obtained, and Equation 26 was used to calculate F1.

In addition to F1, CoQA uses metric exact match (EM) to measure the exact match between the predicted and standard answers. If the two answers are exactly the same, EM = 1; otherwise, EM = 0. QuAC introduced the human equivalence score (HEQ) to judge whether the model prediction reached the human average level, that is, whether modeled F1 exceeded or was equal to Human F1, which was measured in percentage. QuAC designed two evaluation metrics, HEQ-Q and HEQ-D, based on questions and dialogs. HEQ-Q and HEQ-D count the proportion of questions and dialogs, respectively, that meet the aforementioned conditions in each round.

### 4.3 Main results

Table 3 lists the experimental results of the background understanding of the Q&A system for the Bert-Base, Distilbert-Base, and KTM models. The first two models are fine-tuned, whereas KTM uses Bert-Base as a large model and Distilbert-Base as a small model. It was trained using the proposed knowledge transfer method. Because neither CoQA nor QuAC disclosed test sets, Table 1 lists only the experimental results of the validation sets.

**Table 3:** Experimental results with regard to background understanding.

**Model**	**Params**	**Training**	**CoQA**		**QuAC**		
	**(M)**	**(Hour)**	**F1**	**EM**	**F1**	**HEQ-Q**	**HEQ-D**
BERT-base	110	27.00^*^	80.5	71.5	64.5	60.4	6.7
DistilBERT-base	66	13.25^*^	75.0	65.8	59.7	55.5	4.7
KTM	66	13.50	78.8	69.5	**65.3**	**61.4**	6.7

* is the sum of training duration of CoQA and QuAC datasets.

The training and prediction time is the time required to complete the corresponding operation of the model on a single Tesla K40m graphics card.

Bold indicates best results.

As BERT-Base possesses twice as many transformer coding layers as Distilbert-Base, the performances of the two models differ substantially. The proposed knowledge transfer method attempts to bridge this gap by enabling small models to perform better than large models, even with fewer parameters. As listed in Table 3, KTM narrowed the gap between the CoQA validation sets from 5.5 F1 to 1.7 F1. In the QuAC verification set, KTM exceeded BERT-Base, thereby increasing the F1 value by 0.8 and the HEQ-Q value by 1.0; HEQ-D is equal to BERT-Base. By comparing KTM and Distilbert-Base models, which have the same structure but different training methods, we found that the indices of KTM are better than DistilBERT-Base, and the gap is evident. The knowledge transfer method is much better than the direct fine-tuning method.

In Table 3, the sum of the time for one round of training on both datasets is 27 h for BERT-base and 13.25 h for DistilBERT-base. KTM is trained for both datasets together, and the time for one iteration is 13.5 h, which is close to DistilBERT-base but only half of the BERT-base. It can be observed that in terms of training time, knowledge transfer is the same as direct fine-tuning, without increasing the complexity of training. In addition, KTM is consistent with Distilbert-Base but lower than BERT-Base in terms of memory footprint and predicted speed owing to the same model structure.

In summary, the performance of the knowledge transfer model is close to or better than that of the large model, whose scale is approximately twice as large under the condition of low resource consumption, thereby achieving an effective situation of resource consumption and performance.

## 5 Analysis and discussion

To deeply analyze the utility of knowledge transfer, first, the ablation experiment analyzed the contribution of various strategies to the performance of the knowledge transfer model. Subsequently, the impact of knowledge distillation and fine-tuning of similar tasks were analyzed. Finally, the advantages and disadvantages of the knowledge transfer methods were summarized by comparing the four aspects: question type, answer type, answer span length, and dialog rounds.

### 5.1 Ablation study

The main idea of the ablation experiment is to remove one of the above strategies for model training, thereby obtaining KTM w/o KD, KTM w/o QuAC (training CoQA separately), KTM w/o CoQA (training QuAC separately), KTM w/o Four models, such as SQuAD, and then compare the F1 value with KTM. The larger the gap, the greater the impact and contribution are.

The ablation results are listed in Table 4. We found that knowledge distillation considerably affects QuAC, CoQA is influenced primarily by SQuAD fine-tuning, and the effect of co-learning between the two datasets is negligible. After analysis, the reason for the small effect of co-learning may be that the two data sets differently deal with general questions: CoQA uses “yes” and “no” for answers, whereas QuAC uses background text for answers. The different processing modes of the two models lead to the failure of unified cognition in the learning process but increase the noise during training. Thus, by skipping co-learning, the model can still achieve better performance.

**Table 4:** Background understanding ablation experiment results.

**Model**	**CoQA**		**QuAC**	
	**F1**	Δ**F1**	**F1**	Δ**F1**
KTM	78.8	—	65.3	—
− Knowledge Distillation (KTM w/o KD)	77.4	−1.4	63.3	−2.0
− Co-learning with Homogeneous Datasets	78.8	−0.0	64.9	−0.4
− Similar Task Fine-tuning (KTM w/o SQuAD)	75.9	−2.9	63.5	−1.8

Underline indicates best results.

### 5.2 Influence analysis of knowledge distillation

To analyze the impact of knowledge distillation on the model, we compared the F1 of the three models of BERT-base, KTM, and KTM without KD for each question in the validation set. The former ones, that is, BERT-base and KTM, are larger than the latter one. The results are listed in Table 5, and the last row of the table lists the number of questions for which F1 was raised from l < 1 to 1. However, compared to the problem of F1 = 1, both CoQA and QuAC have a large gap. Knowledge distillation can transfer answer-related knowledge from a large model to a small model; however, this transfer can only improve the part of the performance.

**Table 5:** Statistics of the number of questions whose F1 is improved owing to knowledge distillation.

	**CoQA**	**QuAC**
Number of questions with increased F1	205	244
And answer exactly (F1 = 1)	125	112

**Figure 4:**
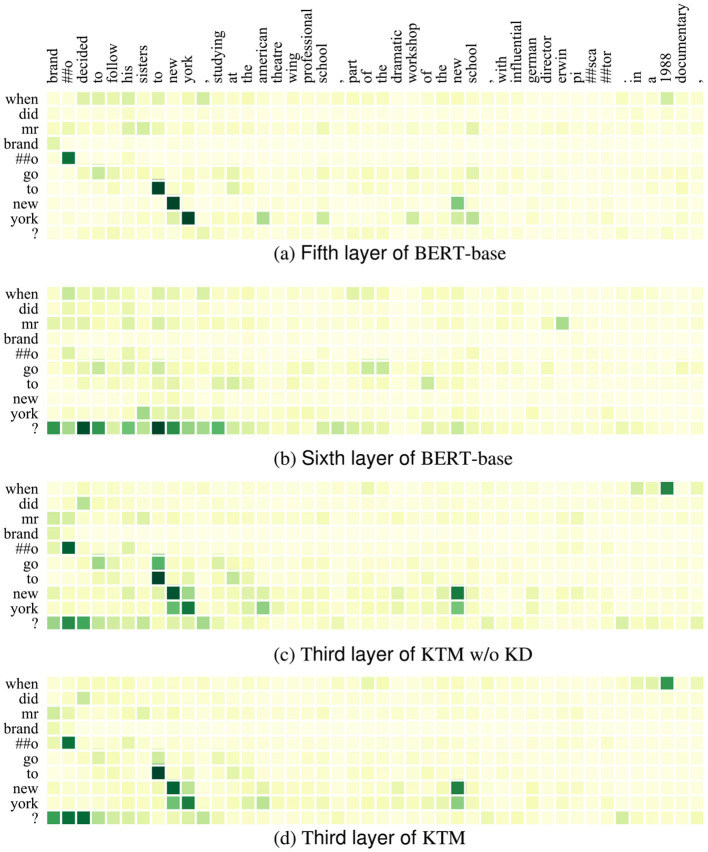
Attention matrix of QuAC example *Q*_1_ and part of background text before and after knowledge distillation. **(a)** Fifth layer of BERT-base. **(b)** Sixth layer of BERT-base. **(c)** Third layer of KTM w/o KD. **(d)** Third layer of KTM.

**Figure 5:**
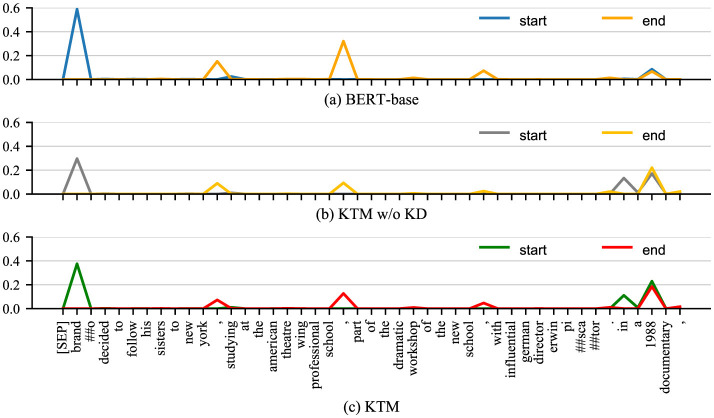
Probability distribution of answer spans for *Q*_1_ in QuAC examples before and after knowledge distillation. **(a)** BERT-base. **(b)** KTM w/o KD. **(c)** KTM.

Figures 4, 5 show the attention matrices of *Q*_1_ and some background text on the three models (BERT-base, KTM w/o KD, and KTM) and the predicted probabilities of the start and end positions of the answer. The “when” in *Q*_1_ is a time-related problem; in Figure 4, KTM w/o KD and KTM focus on “1988”, whereas BERT-base focuses more on “brando” and “to new york.” KTM learned this feature through knowledge distillation; therefore, it increased the attention weight of “brando.” Finally reflected in the span probability $\hat{k}+\hat{l}$ predicted in Figure 1, the KTM w/o KD model lacks attention to “brando” to ensure that the correct answer is in the span “brando ... school,” has a probability value of only 0.391, which is slightly smaller than the probability value of the fragment “1988,” which is 0.392, and finally outputs the wrong answer “1988.” The probabilities of BERT-base with regard to these two spans are 0.910 and 0.153, respectively, which express sufficient affirmation for the correct answer and avoiding the wrong answer. KTM learns this from BERT-base; therefore, it increases the probability value of the span where the correct answer is located at 0.502, whereas the probability value of the span “1988” does not change much to 0.415. As 0.502 > 0.415, KTM, like BERT-base, outputs the correct answer fragment “brando ... school.” Knowledge distillation uses the large model to correct the misunderstanding of the small model.

### 5.3 Analysis of the impact of fine-tuning on similar tasks

Similar to the impact analysis of knowledge distillation, we compared the F1 of each question in the verification set of KTM and KTM W/O SQuAD models, counted the number of the former ones that are greater than the latter ones, and divided them according to the types of answers (Table 6). The purpose of introducing SQuAD fine-tuning in this study was to allow the model to learn in advance how to extract answer spans from the background text; Table 6 lists the SPAN types separately. Thus, we found that SQuAD fine-tuning contributes the most to the SPAN class answer in terms of performance improvement. This demonstrates that SQuAD fine-tuning can allow small models to learn in advance how to extract background snippets from similar machine-reading comprehension tasks, which renders it an indispensable step in knowledge transfer methods.

**Table 6:** Statistics of the number of questions whose F1 has been improved owing to fine-tuning of similar tasks.

	**SPAN answer (percentage)**	**Others**	**Total**
CoQA	435 (86.1%)	70	505
QuAC	410 (88.7%)	52	462

**Figure 6:**
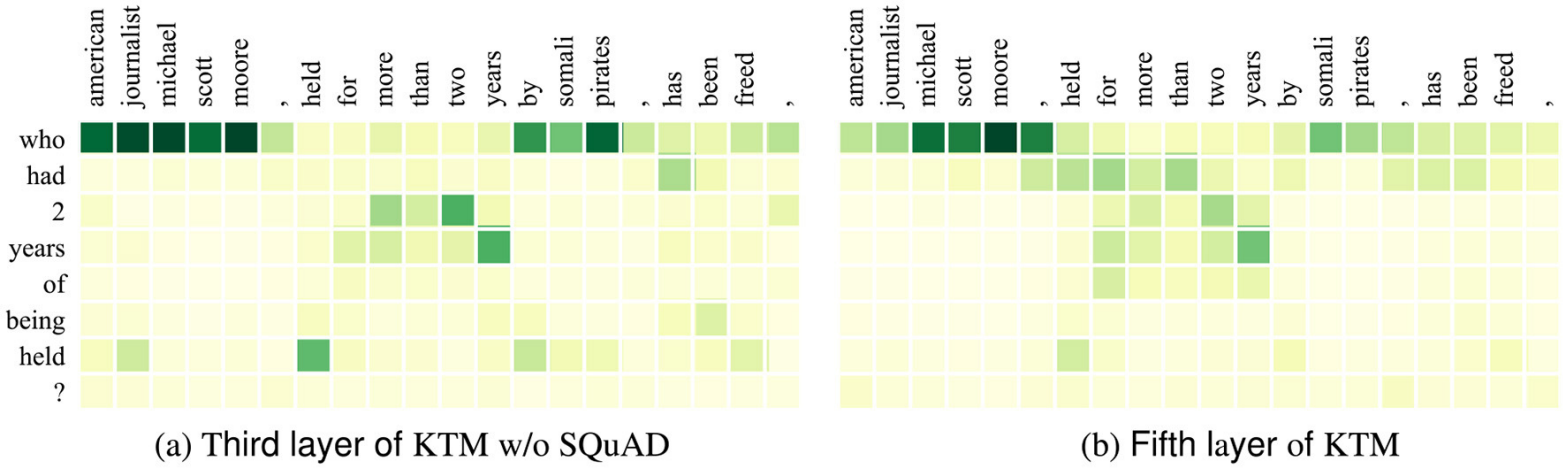
Attention matrix of CoQA example *Q*_1_ and some background text before and after fine-tuning on similar tasks. **(a)** Third layer of KTM w/o SQuAD. **(b)** Fifth layer of KTM.

**Figure 7:**
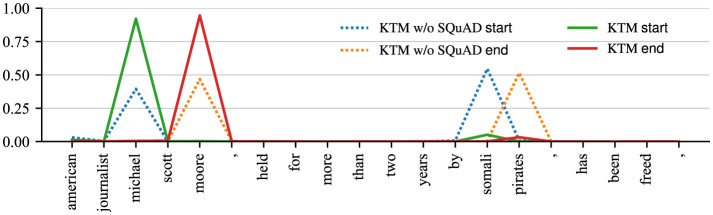
Probability distribution of answer spans for CoQA examples before and after fine-tuning on similar tasks.

Herein, we demonstrate the impact of SQuAD fine-tuning on model decisions using *Q*_1_ in CoQA (Table 1). Figures 6, 7 show the attention matrices of *Q*_1_ and part of the background text on the KTM without SQuAD and KTM models, and the predicted probabilities of the start and end positions of the answer. The “who” in *Q*_1_ is a person-related question; thus, as shown in Figure 6, KTM focuses the “who” attention on the person named entity “michael scott moore,” whereas the KTM w/o SQuAD model on the segment “michael scott moore” and “somali pirates” has concerns. Finally, while making a decision based on the span probability $\hat{k}+\hat{l}$, as shown in Figure 1, the KTM w/o SQuAD model makes a judgment that both spans may be the answers, and as the former probability value (0.864) is less than the latter probability value (1.063); thus, the wrong answer “somali pirates” is the output. As KTM learned the pattern of “who” and person's name from SQuAD, knowing “michael scott moore” was the only correct answer, thereby giving this clip a very high degree of confidence. This demonstrates the positive significance of SQuAD fine-tuning, which makes the extraction of answer spans more accurate.

### 5.4 Compare by type of problem

**Figure 8:**
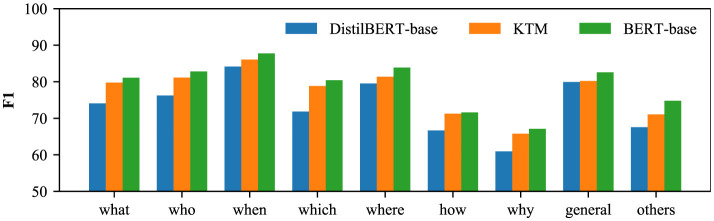
F1 of each problem type in CoQA.

**Figure 9:**
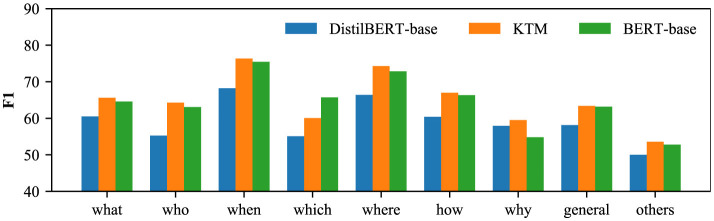
F1 of each problem type in QuAC.

To analyze the impact of knowledge transfer on different types of questions, we divide the questions into “what,” “who,” “when,” “which,” “where,” “how,” “why,” “general” according to the question words and “others” (nine categories). Herein, “general” refers to a general interrogative sentence, and when the question does not meet the first eight categories, it is assigned to the last “others” category. The comparison results of the three models of DistilBERT-base, KTM, and BERT-base on nine types of problems are shown in Figures 8, 9. From the perspective of CoQA, KTM surpasses DistilBERT-base of the same size in all the nine categories of questions, and on “how” category questions, KTM is closest to the large model BERT-base. However, in the “general” category, the performance of KTM has not significantly improved, and Section 5.5 discusses more on this. In terms of QuAC, KTM outperformed Distilbert-Base for all the problem types and BERT-Base for all the types except for “which.” In conclusion, knowledge transfer positively affects the problem types.

### 5.5 Comparison by type of answer

Based on whether the answer exists and general question is answered in the form of yes/no, this study divides CoQA answers into SPAN, YES, NO, and UNANS types and divides QuAC answers into SPAN and UNANS types. When making predictions, the model first determines the answer type and then generates a specific answer text. Therefore, it is necessary to analyze the impact of knowledge transfer on performance by classifying answer types.

**Figure 10:**
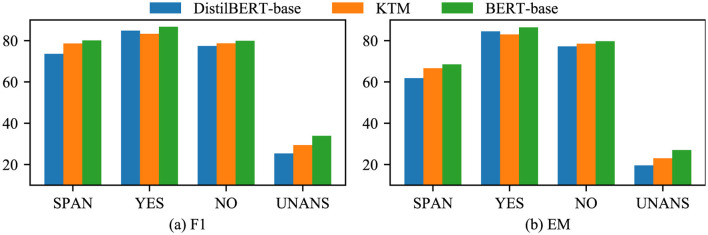
Experimental results of each answer type in CoQA. **(a)** F1. **(b)** EM.

First, we compared the results of the three models: DistilBERT-base, KTM, and BERT-base on CoQA (Figure 10). The performance of the KTM significantly improved based on the SPAN and UNANS answers, followed by NO answers. For the YES answers, the performance declined.

**Figure 11:**
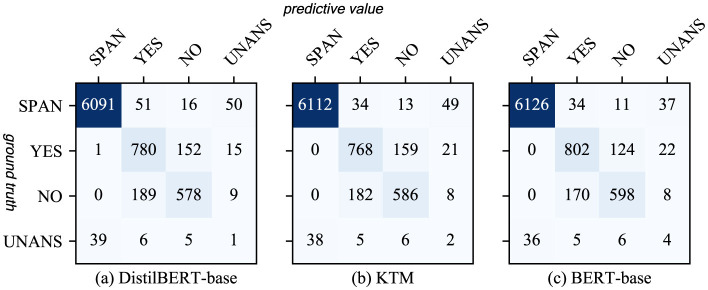
Confusion matrix for answer type discrimination in CoQA. **(a)** DistilBERT-base. **(b)** KTM. **(c)** BERT-base.

Therefore, we analyzed the confusion matrix of answer type discrimination (Figure 11) and found that BERTert-Base misjudged YES as UNANS for a higher number of samples than Distilbert-Base. The KTM retained this feature and misjudged a similar number of YES samples to UNANS. As the YES samples in the training set are fewer than the NO samples, the model is biased, thereby causing another part of the YES samples in the validation set to be misjudged as NO. Therefore, the performance of the KTM with regard to the YES class answer of the CoQA validation set is not at par with DistilBERT-base.

**Figure 12:**
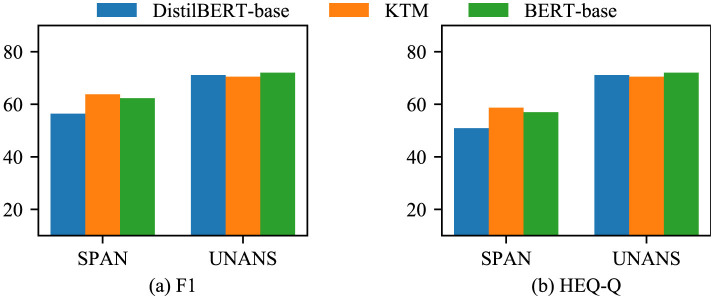
Experimental results of each answer type with regard to QuAC. **(a)** F1. **(b)** HEQ-Q.

**Figure 13:**
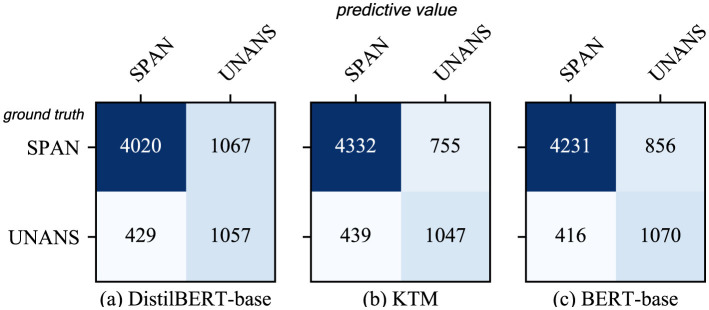
Confusion matrix for answer type discrimination in QuAC. **(a)** DistilBERT-base. **(b)** KTM. **(c)** BERT-base.

Figure 12 shows a comparison of the results of the three models with regard to QuAC. The overall performance of the KTM surpassing the BERT-base is owing to the improvement in the performance of the SPAN-type answers, whereas for the UNANS-type answers, the situation is the same as that of the YES-type answers with regard to CoQA, thus degrading the performance. Further analysis of the answer-type discriminant confusion matrix, as shown in Figure 13, demonstrates that the performance drop is caused by the misclassification of UNANS samples as SPAN. The underlying reason may be that the sample imbalance causes the model prediction to be biased toward SPAN, which is evident from the misjudgment rate (the UNANS misjudgment rate is approximately two times that of SPAN).

Compared with direct fine-tuning, knowledge transfer is beneficial to the performance improvement of different answer types. However, owing to the misjudgment of the large model and the problem of unbalanced samples, in few cases, the performance of a certain type of answer will slightly decrease; however, the overall impact will be negligible.

### 5.6 Compare by length of span

**Figure 14:**
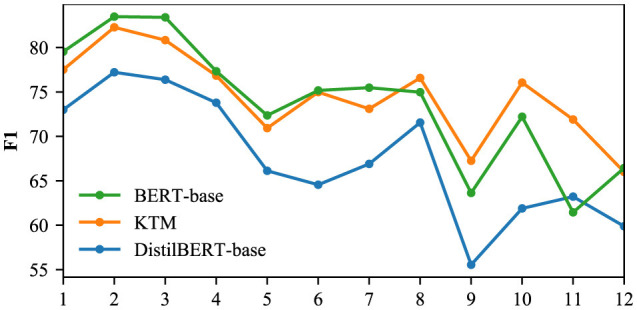
F1 for different answer span lengths in CoQA.

**Figure 15:**
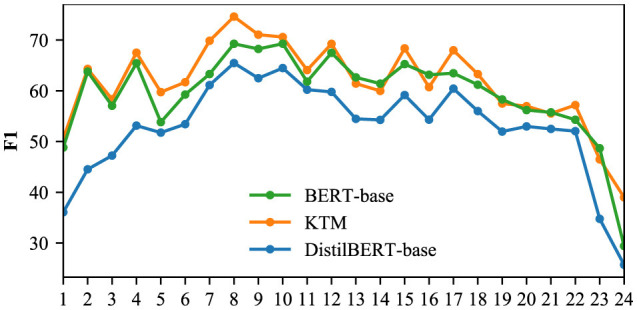
F1 for different answer span lengths in the QuAC.

The SPAN-type answers in the CoQA and QuAC validation sets were filtered out, and the F1 of the three models of DistilBERT-base, KTM, and BERT-base was aggregated by span length; the results are shown in Figures 14, 15. As each question in the validation set corresponds to multiple optional answers, when calculating the length of the answer span for each question, the average is rounded off. After processing, the CoQA and QuAC answer span lengths ranged from 1 to 19 and from 1 to 28, respectively. Although the maximum length of CoQA answer fragments can reach 19, the median is only 2, and text spans with a length of i <12 account for 99.7%. Figure 14 shows the comparison of the results. Similarly, 99.7% of the answer spans in the QuAC validation set were <25 in length, and only these results were compared (Figure 15).

The KTM curve in Figure 14 is close to the BERT-base, and after the length span of eight, KTM surpasses the BERT-base. For this phenomenon, we explain that QuAC answer spans are longer than CoQA, and KTM masters the answering skills of long spans based on the joint learning of the two datasets. For 8–11 in Figure 15, KTM surpasses BERT-base, which further proves that KTM possesses advantages in answering long spans. In addition, the overall KTM curve in Figure 15 is above BERT-base. On QuAC, the overall performance of KTM surpasses that of the large-model BERT-base and is valid for answer spans of all lengths. As shown in Figures 14, 15, we can conclude that compared with direct fine-tuning, knowledge transfer improves the performance of all answer spans of length, particularly for answer spans of 8–11, which exceeds the large model and significantly improves.

### 5.7 Compare by dialogue rounds

A complete interactive Q&A comprises multiple rounds of questioning and answering; therefore, this section attempts to analyze the knowledge transfer utility based on the perspective of dialogue rounds and intends to answer the following questions. (1) How does knowledge transfer occur in different rounds of dialogue? Compared with direct fine-tuning, how much has the performance improved? How much is the difference compared to the big model? (2) As the number of dialogue turns increases, thus gradually decreasing the performance, can knowledge transfer improve this problem?

**Figure 16:**
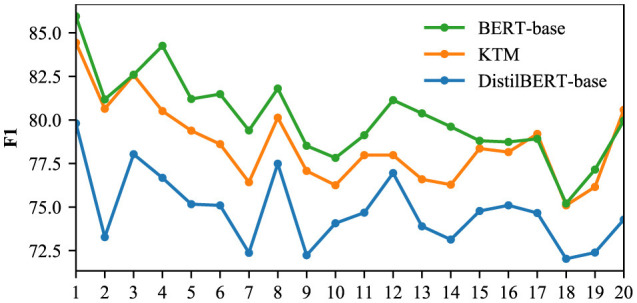
F1 for different dialogue rounds in the CoQA.

**Figure 17:**
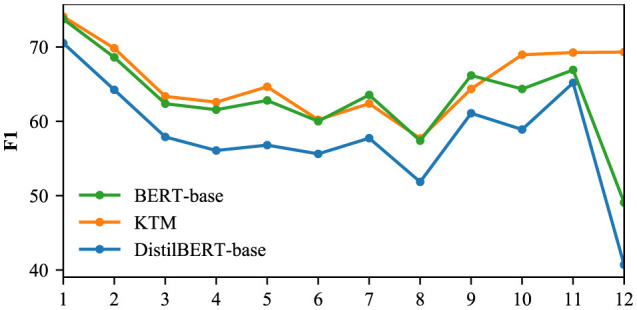
F1 for different dialogue rounds in the QuAC.

We used the CoQA and QuAC validation sets to compare the F1 of the DistilBERT-base, KTM, and BERT-base models for different dialogue rounds, and the results are shown in Figures 16, 17. The CoQA dialogue rounds were distributed between 1 and 25, with an average value of 15.97. There were 11 rounds with more than 20 rounds, accounting for only 2.2% of the total. Therefore, Figure 1 only compares F1 of rounds 1 to 20. The QuAC validation set has 1,000 complete interactive Q&As, with all dialogue turns distributed between 1 and 12. Figure 17 compares F1 for all the rounds.

The KTM curve in Figure 16 is located between BERT-base and DistilBERT-base. Compared with the direct fine-tuning DistilBERT-base of the same scale, the performance of KTM is considerably improved; however, compared with the large model BERT-base, KTM still has a large gap. With an increase in dialogue rounds, all three curves exhibit a trend of fluctuation and decline. The performance of knowledge transfer is consistent with that of direct fine-tuning and fails to address the problem of performance degradation. Figure 17 shows different performances. The KTM curve is slightly higher than that of BERT-base, and in the later stage of interaction (10–12 rounds), when the performance of DistilBERT-base and BERT-base decreases, the KTM trained by knowledge transfer maintains excellent performance. In the QuAC validation set, knowledge transfer significantly improves the performance of all the rounds, which renders it equivalent with or allows it to surpass the large model. Moreover, knowledge transfer has been successful in improving the performance of deep dialogue rounds.

Based on the aforementioned analysis, we have the following answers to the two questions raised at the beginning of this section. In all the dialogue rounds, the models trained by knowledge transfer are better than those trained by direct fine-tuning, and the improvement is significant. With the gap with large models and whether they can improve the performance degradation of deep dialogue rounds, different datasets have different performances.

## 6 Conclusion

This study investigates small pre-trained models for background understanding in automated Q&A. The main work includes a comprehensive comparison of the computing time and performance differences of mainstream pre-training models in the task of Q&A background understanding. A small-scale pre-training model suitable for interactive Q&A background understanding is proposed, and strategies such as knowledge distillation, co-learning of similar data sets, and fine-tuning of similar tasks are used to realize a variety of knowledge transfer and make the performance of small models with low resource consumption, comparable or surpassed by large models. In general, knowledge distillation, co-learning on similar datasets, and fine-tuning on similar tasks play their respective roles in the process of knowledge transfer and contribute to model performance to varying degrees. In future work, we will continue to explore a unified question processing method to enhance the influence of co-learning.

## Data Availability

The original contributions presented in the study are included in the article/supplementary material, further inquiries can be directed to the corresponding author.
